# Safety of pulsed field ablation in more than 17,000 patients with atrial fibrillation in the MANIFEST-17K study

**DOI:** 10.1038/s41591-024-03114-3

**Published:** 2024-07-08

**Authors:** Emmanuel Ekanem, Petr Neuzil, Tobias Reichlin, Joseph Kautzner, Pepijn van der Voort, Pierre Jais, Gian-Battista Chierchia, Alan Bulava, Yuri Blaauw, Tomas Skala, Martin Fiala, Mattias Duytschaever, Gabor Szeplaki, Boris Schmidt, Grégoire Massoullie, Kars Neven, Olivier Thomas, Johan Vijgen, Estelle Gandjbakhch, Daniel Scherr, Arne Johannessen, David Keane, Serge Boveda, Philippe Maury, Ignacio García-Bolao, Ante Anic, Peter Steen Hansen, Franck Raczka, Antoine Lepillier, Yves Guyomar, Dhiraj Gupta, Jurren Van Opstal, Pascal Defaye, Christian Sticherling, Philipp Sommer, Pavel Kucera, Joaquin Osca, Fariborz Tabrizi, Antoine Roux, Michael Gramlich, Stefano Bianchi, Pedro Adragão, Francesco Solimene, Claudio Tondo, Antonio Dello Russo, Jürgen Schreieck, Armin Luik, Obaida Rana, Gerrit Frommeyer, Frédéric Anselme, Ingo Kreis, Raphael Rosso, Andreas Metzner, Laszlo Geller, Samuel H. Baldinger, Angel Ferrero, Stephan Willems, Andreas Goette, Greg Mellor, Shibu Mathew, Lukasz Szumowski, Roland Tilz, Saverio Iacopino, Peter Karl Jacobsen, Andrikopoulos George, Pavel Osmancik, Stefan Spitzer, Richard Balasubramaniam, Abdul Shokor Parwani, Thomas Deneke, Andrzej Glowniak, Antonio Rossillo, Helmut Pürerfellner, David Duncker, Peter Reil, Thomas Arentz, Daniel Steven, Juan José Olalla, Jonas S. S. G. de Jong, Reza Wakili, Selim Abbey, Gottschling Timo, Antonio Asso, Tom Wong, Bertrand Pierre, Niels Christian Ewertsen, Leonard Bergau, Cristina Lozano-Granero, Maximo Rivero, Alexander Breitenstein, Jaakko Inkovaara, Samir Fareh, Decebal Gabriel Latcu, Dominik Linz, Patrick Müller, Javier Ramos-Maqueda, Thomas Beiert, Sakis Themistoclakis, Dirk Grosse Meininghaus, Günter Stix, Stylianos Tzeis, Jakub Baran, Henrik Almroth, Daniel Rodriguez Munoz, João de Sousa, Michalis Efremidis, Pawel Balsam, Jan Petru, Thomas Küffer, Petr Peichl, Lukas Dekker, Domenico G. Della Rocca, Ondrej Moravec, Moritoshi Funasako, Sebastien Knecht, Gael Jauvert, Julian Chun, Romain Eschalier, Anna Füting, Alexandre Zhao, Pieter Koopman, Mikael Laredo, Martin Manninger, Jim Hansen, Daniel O’Hare, Anne Rollin, Zrinka Jurisic, Thomas Fink, Corentin Chaumont, Andreas Rillig, Melanie Gunawerdene, Claire Martin, Bettina Kirstein, Karin Nentwich, Heiko Lehrmann, Arian Sultan, Jan Bohnen, Mohit K. Turagam, Vivek Y. Reddy

**Affiliations:** 1 Icahn School of Medicine at Mount Sinai, New York, NY USA; 2 Winchester Cardiology and Vascular Medicine, Winchester Medical Center Valley Health, Winchester, VA USA; 3 Homolka Hospital, Prague, Czech Republic; 4 Department of Cardiology, Inselspital, Bern University Hospital, University of Bern, Bern, Switzerland; 5 IKEM, Prague, Czech Republic; 6 Catharina Ziekenhuis Eindhoven, Eindhoven, the Netherlands; 7 IHU LIRYC, CHU Bordeaux, University of Bordeaux, Bordeaux, France; 8 Heart Rhythm Management Centre, Postgraduate Program in Cardiac Electrophysiology and Pacing, Universitair Ziekenhuis Brussel, Vrije Universiteit Brussel, European Reference Networks Guard-Heart, Brussels, Belgium; 9 Ceske Budejovice Hospital and Faculty of Health and Social Sciences, University of South Bohemia in Ceske Budejovice, Ceske Budejovice, Czech Republic; 10 Universitair Medish Groningen, Groningen, the Netherlands; 11 University Hospital Olomouc, Olomouc, Czech Republic; 12 Neuron Medical, Brno, Czech Republic; 13 Az sint jan Hospital, Bruges, Belgium; 14 Atrial Fibrillation Institute, Mater Private Hospital, Dublin, Ireland; 15 Cardiovascular Research Institute, Royal College of Surgeons in Ireland, Dublin, Ireland; 16 Cardioangiologisches Centrum Bethanien, Frankfurt, Germany; 17 Clermont Ferrand university hospital, Clermont-Ferrand, France; 18 Department of Electrophysiology, Alfried Krupp Hospital, Essen, Germany; 19 Department of Medicine, Witten/Herdecke University, Witten, Germany; 20 GHP Ambroise Paré Hartmann, Neuilly sur Seine, France; 21 Jessa Hospitals, Hasselt, Belgium; 22 Sorbonne Université, APHP, Pitié-Salpêtrière Hospital, Institute of Cardiology, ICAN Institute for Cardiometabolism and Nutrition, Paris, France; 23 Medical University of Graz, Graz, Austria; 24 Copenhagen University Hospital Gentofte, Gentofte, Denmark; 25 Black Rock Clinic, Dublin, Ireland; 26 Heart Rhythm Management Department, Clinique Pasteur, Toulouse, France; 27 University of Brussels VUB, Jette Brussels, Belgium; 28 University Hospital Rangueil, Toulouse, France; 29 Clinica Universidad de Navarra, University of Navarra, Pamplona, Spain; 30 Instituto de Investigación Sanitaria de Navarra, Pamplona, Spain; 31 University Hospital Center Split, Split, Croatia; 32 Privathospitalet mølholm a/s, Vejle, Denmark; 33 Clinique du Millenaire, Montpellier, France; 34 Centre Cardiologique du Nord, Saint-Denis, France; 35 GHICL Hôpital Saint Philibert, Saint Philibert, France; 36 Liverpool Heart and Chest Hospital, Liverpool, UK; 37 Medisch Spectrum Twente, Enschede, the Netherlands; 38 Cardiology Department, Grenoble Alpes University Hospital and University, Grenoble, France; 39 University Hospital Basel, Basel, Switzerland; 40 Heart and Diabetes Center NRW, Ruhr University Bochum, Bochum, Germany; 41 Regional Hospital Liberec, Liberec, Czech Republic; 42 Polytechnic and University La Fe Hospital, Valencia, Spain; 43 Capio Arytmi center, Stockholm, Sweden; 44 Pole Sante Republique Elsan, Clermont-Ferrand, France; 45 Uniklinikum RWTH Aachen, Department of Cardiology, Aachen, Germany; 46 Ospedale Isola Tiberina Gemelli, Rome, Italy; 47 Hospital de Santa Cruz, Carnaxide, Portugal; 48 Montevergine Clinic (AV), Mercogliano, Italy; 49 Department of Biomedical, Surgical and Dental Sciences, University of Milan, Milan, Italy; 50 Department of Biomedical Science and Public Health, UNIVPM, Ancona, Italy; 51 Arrhythmology Clinic Department, Azienda Ospedaliera Universitaria delle Marche, Ancona, Italy; 52 Medical University of Tuebingen, Tuebingen, Germany; 53 Städtisches Klinikum Karlsruhe, Karlsruhe, Germany; 54 Universitätsklinikum Düsseldorf, Düsseldorf, Germany; 55 Clinic for Cardiology II, Electrophysiology, University of Münster, Münster, Germany; 56 Rouen Hospital, Rouen, France; 57 St. Johannes Hospital Dortmund, Dortmund, Germany; 58 Tel Aviv Sourasky Medical Center, Tel Aviv, Israel; 59 University Heart and Vascular Center, Hamburg, Germany; 60 Semmelweis University, Cardiovascular Center, Budapest, Hungary; 61 Hirslanden Klinik Beau-Site, Bern, Switzerland; 62 Hospital Clínico Universitario de Valencia, Valencia, Spain; 63 Asklepios Hospital St. Georg, Hamburg, Germany; 64 Department of Cardiology and Intensive Care Medicine, St. Vincenz-Hospital, Paderborn, Germany; 65 MAESTRIA Consortium at AFNET, Münster, Germany; 66 Otto-von-Guericke University, Magdeburg, Germany; 67 Cardiology Department, Royal Papworth Hospital, Cambridge, UK; 68 Universitätsklinikum essen, Essen, Germany; 69 National Institute of Cardiology, Warsaw, Poland; 70 Department of Rhythmology, University Heart Center Lübeck, University Hospital Schleswig-Holstein, Lübeck, Germany; 71 German Center for Cardiovascular Research, Partner Site Lübeck, Lübeck, Germany; 72 Maria Cecilia Hospital, Cotignola, Italy; 73 Rigshospitalet, University Hospital of Copenhagen, Copenhagen, Denmark; 74 Henry Dunant Hospital, Athens, Greece; 75 FNKV, Prague, Czech Republic; 76 Praxisklinik Herz und Gefäße, Dresden, Germany; 77 NHSSC Royal Bournemouth Hospital, Bournemouth, UK; 78 Campus Virchow Klinikum, Deutsches Herzzentrum der Charité, Berlin, Germany; 79 Heart Center Bad Neustadt, Neustadt, Germany; 80 Department of Cardiology, Medical University of Lublin, Lublin, Poland; 81 Department of Cardiology, San Bortolo Hospital, Vicenza, Italy; 82 Ordensklinikum Linz Elisabethinen, Linz, Austria; 83 Hannover Heart Rhythm Center, Department of Cardiology and Angiology, Hannover Medical School, Hannover, Germany; 84 Klinikum Ingolstadt, Ingolstadt, Germany; 85 Universitätsklinikum Freiburg, Freiburg, Germany; 86 Universitätsklinikum Köln AöR, Köln, Germany; 87 Department for Electrophysiology, Heart Center University Cologne, Cologne, Germany; 88 Arrhytmia Service, Cardiology Department, University Hospital Marqués de Valdecilla, Santander, Spain; 89 OLVG, Amsterdam, the Netherlands; 90 University Duisburg-Essen, Duisburg, Germany; 91 University Hospital Frankfurt, Goethe University Frankfurt, Frankfurt, Germany; 92 L’Hôpital Privé du Confluent, Nantes, France; 93 Christliches Klinikum Unna, Unna, Germany; 94 Hospital Quironsalud, Zaragoza, Spain; 95 Royal Brompton and Harefield Hospitals, Guy’s and St Thomas’ NHS Foundation Trust, King’s College and Imperial College, London, UK; 96 CHRU de Tours, Hôpital Trousseau, Saint-Avertin, France; 97 Vivantes Klinikum Am Urban, Berlin, Germany; 98 Göttingen University Medical Center, Göttingen, Germany; 99 Arrhythmia Unit, Cardiology Department, University Hospital Ramón y Cajal, Madrid, Spain; 100 Ziekenhuis Oost Limburg, Genk, Belgium; 101 Kantonsspital Baden AG, Baden, Switzerland; 102 Tampere Heart Hospital, Tampere University Hospital, Tampere, Finland; 103 Hopital de la Croix Rousse Nord, Nord, France; 104 Centre Hospitalier Princesse Grace, Jardin Exotico, Monaco; 105 Department of Cardiology, Cardiovascular Research Institute Maastricht (CARIM), Maastricht University Medical Centre, Maastricht, the Netherlands; 106 Faculty of Health and Medical Sciences, Department of Biomedical Sciences, University of Copenhagen, Copenhagen, Denmark; 107 Knappschaftskrankenhaus Recklinghausen, Recklinghausen, Germany; 108 Arrhythmias Unit, Cardiology Department, Lozano Blesa Clinical University Hospital, Zaragoza, Spain; 109 Aragón Health Research Institute, Zaragoza, Spain; 110 Heart Center Bonn, Department of Internal Medicine II, University Hospital Bonn, Bonn, Germany; 111 Ospedale dell’Angelo, Mestre, Italy; 112 Carl-Thiem-Klinikum Cottbus, Cottbus, Germany; 113 Allgemeines Krankenhaus Universitätsklinik Wien, Wien, Austria; 114 MITERA Hospital, Marousi, Greece; 115 Department of Internal Medicine and Cardiology University Clinical Center, Medical University of Warsaw, Warsaw, Poland; 116 Linköpings University Hospital, Linköping, Sweden; 117 Hospital Doce de Octubre, Madrid, Spain; 118 Arrhythmia Unit, Cardiology Department, Lisbon Academic Medical Center, Santa Maria University Hospital, Lisbon, Portugal; 119 Onassis Cardiac Surgery Center, Department of Cardiac Electrophysiology and Pacing, Athens, Greece; 120 1st Chair and Department of Cardiology, Warsaw Medical University, Warsaw, Poland

**Keywords:** Outcomes research, Atrial fibrillation

## Abstract

Pulsed field ablation (PFA) is an emerging technology for the treatment of atrial fibrillation (AF), for which pre-clinical and early-stage clinical data are suggestive of some degree of preferentiality to myocardial tissue ablation without damage to adjacent structures. Here in the MANIFEST-17K study we assessed the safety of PFA by studying the post-approval use of this treatment modality. Of the 116 centers performing post-approval PFA with a pentaspline catheter, data were received from 106 centers (91.4% participation) regarding 17,642 patients undergoing PFA (mean age 64, 34.7% female, 57.8% paroxysmal AF and 35.2% persistent AF). No esophageal complications, pulmonary vein stenosis or persistent phrenic palsy was reported (transient palsy was reported in 0.06% of patients; 11 of 17,642). Major complications, reported for ~1% of patients (173 of 17,642), were pericardial tamponade (0.36%; 63 of 17,642) and vascular events (0.30%; 53 of 17,642). Stroke was rare (0.12%; 22 of 17,642) and death was even rarer (0.03%; 5 of 17,642). Unexpected complications of PFA were coronary arterial spasm in 0.14% of patients (25 of 17,642) and hemolysis-related acute renal failure necessitating hemodialysis in 0.03% of patients (5 of 17,642). Taken together, these data indicate that PFA demonstrates a favorable safety profile by avoiding much of the collateral damage seen with conventional thermal ablation. PFA has the potential to be transformative for the management of patients with AF.

## Abstract (web summary)

In a post-approval study including more than 17,000 patients on the safety of pulsed field ablation, a new method for treatment of atrial fibrillation, the procedure was found to have a low rate of adverse events but was associated with some unexpected rare complications that will need further study.

## Main

Atrial fibrillation (AF) is the most common sustained heart rhythm disorder, with notable impact on quality of life, morbidity and mortality^1–4^. Catheter ablation using thermal energy is an effective means to treat AF, even as a first-line therapy to improve quality of life and morbidity and even to prevent mortality in heart failure patients^5–7^. Technological evolution in catheter design, mapping and optimization across the spectrum of thermal ablation modalities (radiofrequency/laser/heat or cryothermy/cold) have made positive strides in improving its safety and efficacy.

However, inherent to thermal ablation is the indiscriminate nature of tissue destruction, which can have deleterious consequences on the myocardium and pericardiac structures. While the overall complication rates during thermal ablation have improved over time, there remain residual safety considerations including the risk for pulmonary vein (PV) stenosis, stroke, phrenic nerve palsy and the deadliest complication, atrio-esophageal fistula, which even today has a mortality of ~50% (refs. ^8–10^).

Pulsed field ablation (PFA) is an emerging AF ablation modality with an important degree of preferentiality to myocardial tissue damage. By delivering ultrarapid (microsecond to nanosecond) electrical pulses to generate strong electrical fields, PFA can produce irreversible nanoscale pore formation culminating in cellular death^11,12^. Pre-clinical studies demonstrated no (or little) damage to peri-atrial tissue such as the esophagus and phrenic nerve, and no PV stenosis^13–16^. The first and most extensively investigated PFA catheter is a multi-electrode pentaspline catheter, studied in first-in-human trials for treating either paroxysmal or persistent AF patients in IMPULSE/PEFCAT/PEFCAT2 and PersAFOne, respectively^17–19^. These trials demonstrated the feasibility and safety of PFA for AF ablation in a relatively small cohort of patients (<150) and few operators. Though promising, concerns remained around the safety of this novel ablation modality, particularly in a ‘real-world’ setting with a large volume of patients and operators.

After European regulatory approval (CE mark certification) of the pentaspline PFA catheter in March 2021, the MANIFEST-PF Survey of all AF patients receiving PFA in year 2021 (*n* = 1,758 patients at 24 centers) revealed no esophageal damage or PV stenosis and rare phrenic palsy (<1 in 1,000) with good clinical effectiveness^20–22^. These findings were consistent with the safety observed in the EU-PORIA registry (*n* = 1,233 patients at 7 centers). Additionally, the recently published ADVENT randomized clinical trial demonstrated noninferiority of PFA to conventional thermal ablation (cryothermal or radiofrequency) for efficacy and safety in a cohort of 707 paroxysmal AF patients^23^.

While encouraging, it is important to recognize that: (1) when cryoballoon ablation was first introduced approximately two decades ago, atrio-esophageal fistula formation was observed only after a few thousand patients were treated, and (2) unforeseen PFA-related adverse events (AEs) may only manifest after several thousands of procedures are performed^24–26^. Accordingly, the retrospective MANIFEST-17K study assessed the safety of PFA in the very large cohort of >17,000 patients.

## Results

MANIFEST-17K is a retrospective observational study of centers performing PFA after regulatory approval of the pentaspline PFA catheter (Farawave, Farapulse-Boston Scientific Inc.) with the goal of collecting comprehensive data on the methods and safety of the post-approval clinical use of PFA. An invitation to participate in MANIFEST-17K was sent to all 116 centers performing post-approval clinical cases with this PFA catheter. Institution-level data were obtained on center characteristics, limited patient baseline characteristics, limited procedure parameters, and all AEs. We excluded from this analysis the initial 1,758 patients treated in 2021 by the initial 24 centers (herein referred to as the ‘initial MANIFEST-PF sites’) and previously reported in the MANIFEST-PF survey (the ‘MANIFEST-PF cohort’)^20,21^.

### Baseline characteristics

#### Clinical site characteristics

**Fig. 1:**
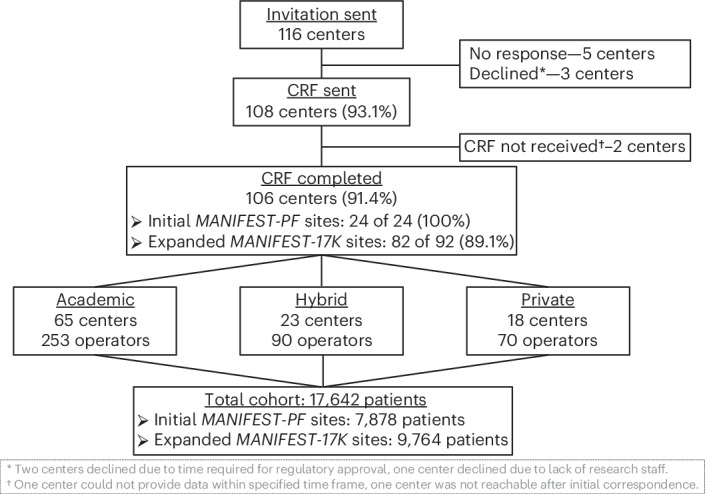
Study center, operator and patient numbers. Shown are the invited and participating centers, along with the number of operators and the number of patients included in the study. CRF, case report form.

Out of 116 centers contacted for participation, a total of 106 centers agreed (91.4% response), including the 24 initial MANIFEST-PF sites, plus 82 of the expanded MANIFEST-17K sites (Fig. 1). Clinical centers were located in 20 countries, 19 in Europe and 1 in Israel. All data forms were considered complete. Of the ten nonparticipating centers, five were not reachable, three declined participation owing to the time required for either local ethics approval or insufficient research staff, and two could not provide the data within the specified time frame.

As shown in Extended Data Table 1, most centers (61.3%) self-classified as academic, 21.7% were hybrid and 17% were private practice. The mean number of operators per center was 3.9 (range 1–11), with an average of 13.6 years in practice (range 3–25 years). The average number of AF ablations performed annually was 590 (range 80–2,000). The date of the first PFA case performed ranged from March 2021 to March 2023, with each site performing an average of 166 PFA cases (range 17–1,277). The average follow-up time was 15 months (range 3–25 months).

As compared to the initial MANIFEST-PF sites (*n* = 24), the expanded MANIFEST-17K sites (*n* = 82) similarly classified themselves as private (18.3% versus 12.5%, respectively, *P* = 0.506) or hybrid (20.7% versus 25%, respectively, *P* = 0.655). The initial MANIFEST-PF sites were higher volume centers for total AF ablation (801 (200–2,000) versus 436 (80–1,200), *P* < 0.001), and for PFA cases (328 (54–1,277) versus 119 (17–472), *P* < 0.001) and, as expected, had earlier exposure to PFA (average first case—June 2021 versus June 2022; Extended Data Table 1).

#### Patient characteristics

The study population included a total of 17,642 patients who underwent PFA between early 2022 to, for most centers, June 2023. The mean age was 64 years (range 11–96 years), of which 34.7% were female (Table 1). The type of AF treated was paroxysmal (57.8%), persistent (35.2%), long-standing persistent AF (5.6%) or, infrequently, atrial flutter/atrial tachycardia (1.4%). The procedures were performed under deep sedation without intubation in 53.1% of the patients.

**Table 1:** Baseline patient characteristics

	Full MANIFEST-17K cohort (*N* = 17,642)
Demographic	
Age (years), mean (minimum–maximum)	64 (11–96)
Female (%)	34.7
Indication for ablation	
Paroxysmal atrial fibrillation (%)	57.8
Persistent atrial fibrillation (%)	35.2
Long-standing persistent atrial fibrillation (%)	5.6
Atrial flutter/atrial tachycardia (%)	1.4
Sedation	
General anesthesia (%)	46.9
Deep sedation/no intubation (%)	53.1

### AEs overview

**Fig. 2:**
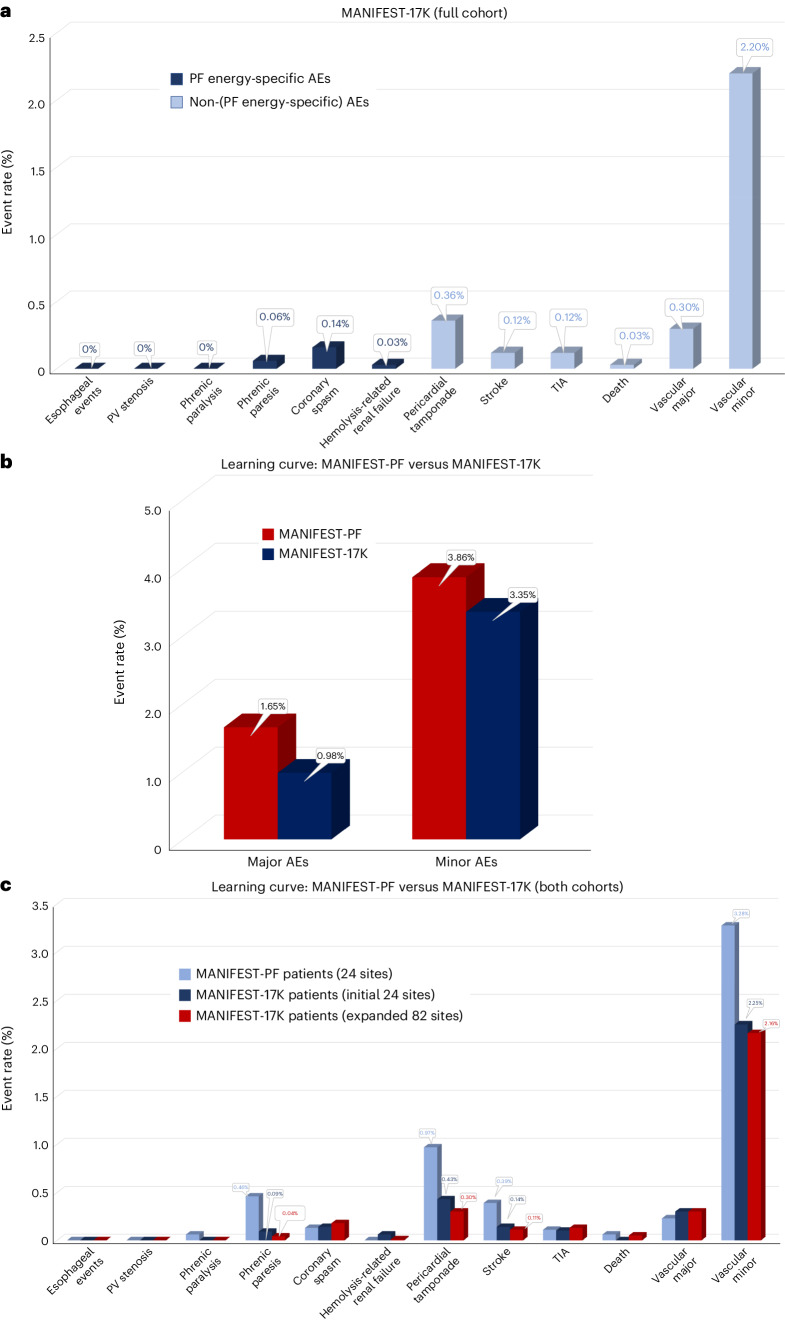
Complication rates. **a**, Shown are the rates of complications, partitioned by relationship to pulsed field energy. Dark blue, complications with some degree of specificity to the energy source; light blue, complications that are more general to catheter ablation procedures. **b**, Shown are aggregated major and minor AE rates as initially reported in the MANIFEST-PF cohort (*n* = 1,758 patients; red bars) and observed in the currently studied MANIFEST-17K cohort (*n* = 17,642 patients; blue bars). **c**, Shown is a comparison of complication rates between those observed in the MANIFEST-PF cohort (light blue) as compared to both subcohorts of MANIFEST-17K. TIA, transient ischemic attack.

As shown in Table 2 and Fig. 2a, in the 17,642 patient cohort, the major complication rate was 0.98%. The most common of these major complications were of vascular origin (0.30%) and pericardial tamponade (0.36%), with the majority of the latter being treated percutaneously (56 of 63, 88.9%) instead of surgically (7 of 63, 11.1%). The remaining major complications included stroke (0.12%) and coronary spasm (0.14%), with mortality being rare at 0.03% (*n* = 5).

**Table 2:** Major and minor complications

	Full patient cohort from all 106 MANIFEST-17K sites^a^ (*N* = 17,642)
Major AEs	173 (0.98%)
Death^b^	5 (0.03%)
Stroke	22 (0.12%)
Esophageal fistula or dysmotility	0 (0%)
Pulmonary vein stenosis	0 (0%)
Phrenic nerve injury (persistent)^c^	0 (0%)
Pericardial tamponade^b^	63 (0.36%)
Percutaneous intervention	56 (0.32%)
Surgical intervention^b^	7 (0.04%)
Vascular complication (with intervention)	53 (0.30%)
Coronary artery spasm	25 (0.14%)
Myocardial infarction	0 (0.0%)
Hemolysis renal failure (hospitalization)	5 (0.03%)
Other (thrombosis)	1 (0.006%)
Minor AEs	567 (3.21%)
Transient ischemic attack	21 (0.12%)
Phrenic nerve injury (transient)^c^	11 (0.06%)
Pericardial effusion (no intervention)	59 (0.33%)
Pericarditis	30 (0.17%)
Vascular complications (no intervention)	388 (2.20%)
Hemolysis renal failure (no hospitalization)	1 (0.006%)
Other complications	57 (0.32%)

^a^The initial 1,758 patients treated in 2021 (and previously reported in the MANIFEST-PF survey) are excluded from this analysis.

^b^One patient requiring surgical intervention for tamponade subsequently died and is thus counted in both categories.

^c^Persistent injury is defined as being present after hospital discharge, while transient injury is defined as having recovered by the time of discharge.

Overall major and minor event rates have been highlighted in bold.

The minor complication rate was 3.21%, composed primarily of vascular complications (2.2%) and, to a lesser extent, pericardial effusion not requiring intervention (0.33%). Other minor complications included transient ischemic attack (0.12%), pericarditis (0.17%) and transient phrenic injury (0.06%)—the last defined as phrenic palsy that recovered before hospital discharge.

### PFA energy-specific AEs

There were no post-PFA esophageal complications, including no reported instances of atrio-esophageal fistula formation or dysmotility disorders (Table 2). There were also no instances of pulmonary vein stenosis.

#### Phrenic nerve

Persistent phrenic nerve injury was not reported. However, transient phrenic paresis was reported in 11 patients (0.06%), with patients recovering diaphragmatic function by the next day.

#### Coronary spasm

There was evidence of coronary arterial spasm in 25 patients (0.14%). The majority of these cases were proximity-related spasm (22 of 25, 88%), and the remaining were instances of generalized/remote spasm (Table 3). Electrocardiogram (EKG) changes were observed in most patients (23 of 25; 92%), hypotension was observed in 5 cases (20%) and intravenous or intracoronary nitroglycerin was administered in 21 cases (84%). Clinical sequelae were reported in four cases (16%): (1) one patient developed atrioventricular block and ventricular fibrillation during PFA of the cavotricuspid isthmus (CTI), prompting resuscitation and defibrillation^27^, (2) two patients developed chest pain in the post-procedure recovery area, with both promptly resolved with nitroglycerin, and (3) one patient developed anterior ST elevation, polymorphic premature ventricular contractions and subsequent ventricular fibrillation after PFA at the right inferior PV—prompting resuscitation, defibrillation and intravenous nitroglycerin administration.

**Table 3:** Coronary artery spasm

	Coronary spasm (*N* = 25)
Type of spasm:	
Proximity-related spasm^a^	22 (88%)
Generalized spasm^b^	3 (12%)
EKG changes	23 (92%)
Hypotension	5 (20%)
Clinical sequelae	4 (16%)
Chest pain	2 (8%)
Ventricular fibrillation	2 (8%)
Intravenous nitroglycerin administered	21 (84%)

^a^Spasm occurring during PFA adjacent to a coronary artery, either during mitral isthmus or CTI ablation.

^b^Spasm occurring during conventional PV application remote from the location of a coronary artery.

#### Hemolysis-related renal failure

**Extended Data Fig. 1:**
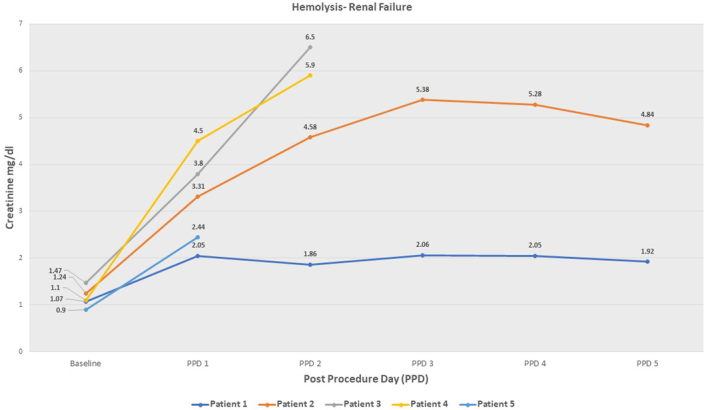
Hemolysis-Renal Failure. Shown are the creatinine trends for the five patients with hemolysis related renal failure.

Hemolysis with resultant acute renal failure occurred in five patients (0.03%). The creatinine level increased by 100% by the next post-procedure day in all patients, with a peak creatinine of 6.5 mg dl^−1^ in one patient (Extended Data Fig. 1). Symptomatology included hemoglobinuria, nausea and oliguria, beginning either immediately post-procedure or the next day. Three patients had normal creatinine levels at baseline, while two patients had baseline elevation (1.2 and 1.5 mg dl^−1^). For all patients, transient hemodialysis significantly improved renal function by the time of hospital discharge.

All five patients had received PFA for persistent AF, with a complex lesion set including pulmonary vein isolation (PVI), posterior wall ablation, mitral isthmus and CTI lines. Importantly, an extensive number of PF applications (143 ± 27 per procedure) had been placed (patient details in Extended Data Table 2).

#### Hemolysis

One other patient was reported as having hemolysis, but without kidney injury (Table 2). In addition, there were several patients at one other center reported to have ‘dark urine or hemoglobinuria’ in either the immediate post-procedure setting or the next day. However, there was no reported kidney injury or drop in the red cell count.

### Non-PFA energy-specific AEs

#### Pericardial tamponade

Pericardial tamponade occurred in 63 patients (0.36%), with the majority (*n* = 56, 0.32%; or 56 of 63, 88.9%) managed with percutaneous pericardiocentesis. Surgery was required in the remaining seven patients (0.04%), of which details were available for four: (1) right atrial appendage injury related to the guidewire for transeptal puncture, (2) laceration of the left atrium (LA) and right inferior pulmonary vein, (3) left ventricular perforation with the PFA catheter while trying to probe the left inferior pulmonary vein, and (4) perforation/tamponade with emergent sternotomy and repair but irreversible brain damage culminating in death (see below).

#### Stroke

Stroke occurred in 22 patients (0.12%). In a root cause analysis including 16 of these patients, the most common putative cause was catheter exchanges/sheath management in 4 cases (Extended Data Table 3). Other putative contributory causes were an activated clotting time (ACT) <300 and interruption of anticoagulation in each of two patients, and uncontrolled hypertension in one patient. No definitive cause was identified in seven cases. None of these strokes culminated in death.

In a subset of the MANIFEST-17K cohort, 96 patients at eight clinical sites underwent routine post-procedural brain magnetic resonance imaging (MRI) to assess for asymptomatic lesions. Such asymptomatic MRI abnormalities were observed in nine patients (9.4%).

#### Vascular complications

Vascular complications occurred in 2.5% of patients, with most being minor complications (2.2%) not requiring intervention (Table 2). As shown in Extended Data Table 4, the most commonly observed vascular complications were hematomas (1.84%). The cohort was stratified by centers that did versus did not routinely use ultrasound guidance for vascular access: 55 sites versus 48 sites, respectively (data on ultrasound usage were not reported by 3 sites). The sites routinely using ultrasound were more likely to be classified as academic (70.9% versus 52.1%, *P* = 0.037) and less likely to be private (5.5% versus 29.2%, *P* = 0.0014). The clinical site characteristics and baseline patient characteristics were similar between both groups, including the number of PFA cases per site (182 (range 17–707) versus 151 (18–1,277), *P* = 0.393). However, the rate of major vascular complications was significantly higher in the group not routinely using ultrasound guidance (0.50% versus 0.17%, *P* = 0.046; Extended Data Table 4).

#### Mortality

The mortality rate was 0.03% (*n* = 5) in the MANIFEST-17K cohort. The available relevant details for each patient are listed in Extended Data Table 5. Briefly, two deaths were clearly procedure-related—a cardiac tamponade prompting emergent surgical repair but resulting in irreversible neurological damage and post-procedure cardiogenic shock in a patient with cardiomyopathy and decompensated heart failure. The remaining three deaths occurred at 3, 9 and 30 days post-ablation—two were sudden deaths that were unexplained or in the setting of severe coronary artery disease, respectively, and the last was a noncardiovascular death secondary to a brain hemorrhage.

#### Other complications

Complications categorized as ‘other’ occurred in 57 patients—0.32% of the full cohort. As shown in Table 4, the most common of these was the need for pacemaker implantation, occurring in 0.07% (*n* = 12), with lead dislocation or malfunction in another 0.02% (*n* = 3). Air emboli without clinical sequelae occurred in 0.06% (*n* = 10). Hemoptysis was noted in 0.02% (*n* = 3). Other notable unusual complications included individual cases of Takotsubo, Bell’s palsy and electrical isolation of the left atrial appendage (Extended Data Table 6).

**Table 4:** Other complications

	Other complications *N* = 57 (0.32%)
Pacemaker implant	12 (0.07%)
Air emboli	10 (0.06%)
Lead malfunction/dislocation	3 (0.02%)
Atrioventricular block	3 (0.02%)
Migraine	3 (0.02%)
Hemoptysis	3 (0.02%)
Anesthesia-related hypotension	2 (0.01%)
Heart failure	2 (0.01%)
Pneumonia	2 (0.01%)
Gastritis	2 (0.01%)
Miscellaneous	15 (0.09%)

### Learning curve

The initial 24 sites included in the previously published MANIFEST-PF study were the first sites/operators to utilize PFA for AF ablation after regulatory approval in Europe. There was an overall decrease in the rate of AEs when comparing outcomes from the 1,758 patients from MANIFEST-PF versus these same sites’ experience with the subset of 7,878 patients treated by these sites in MANIFEST-17K. There were trends for reduced rates of both major (1.65% versus 0.98%, *P* = 0.193) and minor (3.86% versus 3.35%, *P* = 0.266) AEs (Fig. 2b). As shown in Fig. 2c and Extended Data Table 6, there were numerical reductions in the rates of cardiac tamponade (0.97% versus 0.43%, *P* = 0.093) and minor vascular complications (3.28% versus 2.25%, *P* = 0.326). There were also numerical reductions in the rates of stroke (0.39% versus 0.14%, *P* = 0.387), transient phrenic nerve paresis (0.46% versus 0.09%, *P* = 0.344) and mortality (0.06% versus 0%, *P* = 0.323), although these did not reach statistical significance. The rate of major vascular complications was not different (0.23% versus 0.30%, *P* = 0.592).

**Extended Data Fig. 2:**
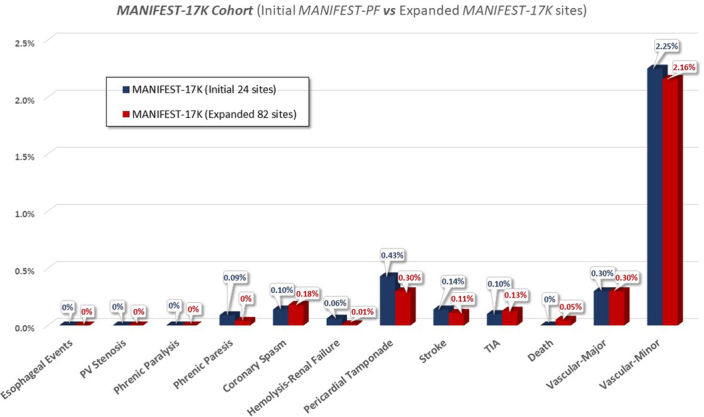
***MANIFEST-17K Cohort*** **(Initial** ***MANIFEST-PF vs*** **Expanded** ***MANIFEST-17K*** **sites**.

In addition to the site-level learning observed at these 24 sites, there was also evidence of community-level global learning: when the AE rates of the initial MANIFEST-PF sites were compared to the expanded MANIFEST-17K sites, there was no significant difference in complication rates (Extended Data Fig. 2). Interestingly, all five deaths in this study occurred in the expanded MANIFEST-17K cohort, while all five cases of hemolysis with renal failure occurred in the initial MANIFEST-PF cohort.

## Discussion

The MANIFEST-17K registry is a multicenter multinational study including 17,642 consecutive unselected AF patients undergoing post-approval PFA with a pentaspline catheter in routine clinical practice. Patients were treated at 106 clinical sites—representing >90% of all centers employing this PFA catheter. The major findings are: (1) there were no esophageal complications, symptomatic PV stenoses or persistent phrenic nerve injury, demonstrating the tissue preferentiality of PFA; (2) the overall rate of non-PFA energy-specific AEs was low, including a major complication rate of 0.98%—primarily pericardial tamponade and vascular complications—and a minor complication rate of 3.21%—primarily of vascular etiology; (3) coronary spasm occurred in 0.14%, primarily proximity-related vasospasm (0.12%), and to a lesser extent, generalized vasospasm (0.02%) during PVI; (4) there was an unexpected finding of hemolysis with associated acute renal failure requiring temporary hemodialysis in five patients (0.03%), although all patients recovered without sequalae; (5) the overall mortality rate was low at 0.03%; and (6) there was evidence of both center-level and global community-level learning with reduced rates of key AEs in the MANIFEST-17K cohort as compared to the previously published MANIFEST-PF cohort.

PFA is being utilized across the spectrum of AF, mostly paroxysmal AF (57.8%), but also persistent AF (35.2%). This is unsurprising given the success of PVI alone in many patients with persistent AF, and especially since posterior wall ablation is relatively easy to perform with PFA. PFA utilization spanned all practice settings, mostly academic centers (61.3%). Patient demographics, average age of 64 (11–96) and 34% female were consistent with routine clinical practice.

For procedural workflow, it is interesting that the use of deep sedation without endotracheal intubation and general anesthesia with intubation was relatively evenly split, slightly in favor of the former: 56.1% versus 43.9%, respectively. In the initial MANIFEST-PF survey, the majority of cases (82.1%) were performed without endotracheal intubation. The reason for this variance is unclear, but perhaps related to better access to anesthesia services, or a desire to minimize diaphragmatic stimulation and cough.

The cohort enrolled in this study, namely 17,642 patients, represents the largest PFA study so far. In this real-world cohort, preferentiality to tissue ablation was demonstrated for PFA, including no esophageal complications, PV stenosis or persistent phrenic nerve injury. This is consistent with prior pre-clinical, observational and randomized clinical studies.

Pre-clinical studies demonstrated the esophageal sparing properties of PFA. In an open chest porcine model, PFA application (200 J) directly atop the esophagus resulted in only intraepithelial vesicles being noted in the esophageal adventitia on day 2, with complete normalization by day 7 (ref. ^13^). In another porcine model, which better approximated the clinical situation, the esophagus was mechanically apposed against the inferior vena cava, from which either radiofrequency ablation (RFA) or PFA was performed. PFA revealed no chronic histopathological esophageal changes, while RFA demonstrated the full spectrum of esophageal injury including deep ulcers, abscesses and fistula formation^15^.

The initial clinical experience, including the initial MANIFEST-PF survey of >1,700 patients, also revealed no esophageal complications. Although promising, cryoballoon ablation was also initially thought to not result in esophageal complications; however, this was proven untrue after a few thousand patients were treated^24–26^. In the POTTER-AF worldwide survey including 553,729 procedures, the incidence of esophageal fistula was 0.025% (RFA, 0.038% (1 in 2,600) and cryoablation, 0.0015% (1 in 66,000)), with an associated mortality rate of 65.8% (ref. ^8^). In this context, it is striking that in the present MANIFEST-17K cohort of >17,000 patients, with no esophageal management strategy employed during procedures, no esophageal complications were observed. These data are entirely consistent with a post-ablation chest MRI study of patients undergoing either PFA (*n* = 18) or thermal ablation (*n* = 23; radiofrequency or cryoballoon) for AF; acute esophageal lesions were observed in 43% of thermal cases, but none with PFA^16^.

Interestingly, at one center, routine post-procedural esophagogastroduodenoscopy was performed in all patients (*n* = 29) undergoing PFA ablation. Imaging identified four cases of esophageal wall edema. However, there was no evidence of ulceration or gastric dysmotility, and no clinical symptoms were reported.

In pre-clinical studies of phrenic nerve injury, only transient (recovering in 30 min) phrenic nerve palsy was observed^14,28,29^. There were no histological changes suggestive of nerve injury. In the MANIFEST-PF registry of 1,568 patients, only 1 patient (0.06%) sustained phrenic nerve injury persisting beyond 1 year^21^. In the randomized ADVENT trial, persistent phrenic nerve injury was observed in 2 of 302 thermal ablation patients (0.7%), as opposed to 0 of 305 PFA patients.

In the present MANIFEST-17K cohort of >17,000 patients, there were no instances of persistent phrenic nerve injury and only 11 cases (0.06%) of transient phrenic nerve injury recovering within a few minutes or by the next day, with the latter nominally less frequently observed than in the initial MANIFEST-PF study (0.46%; *P* = 0.29). It has been postulated that transient phrenic nerve paresis may represent electrical hyperpolarization due to its rapid recovery. Nonetheless, care should be taken to limit excessive PFA in proximity to the phrenic nerve, and routine monitoring of post-PFA diaphragmatic function should be considered.

There were no reported cases of PV stenosis in this MANIFEST-17K cohort. Importantly, this is in the context of a large number of operators (*n* = 413) with varying experience (average 13.6 years, range 3–25 years). Notably, this potential complication was not prospectively defined and routine post-ablation PV imaging was not performed. However, these findings are in line with prior pre-clinical and clinical studies, including a nonrandomized comparison of RFA to PFA: neither PV stenosis nor even PV narrowing was present with PFA, whereas PV stenosis/narrowing was present in 32.5% of patients with RFA^30^. In the randomized ADVENT trial, there was a significant average decrease in the change in PV cross-sectional area 3 months post-ablation with thermal ablation (12.0%) versus no significant decrease with PFA (0.9%; posterior probability of superiority of PFA >99.9%)^23^.

There were 25 cases (0.14%) of coronary spasm: (1) most were proximity-related (*n* = 23, 88%), that is, occurring during PFA adjacent to a coronary artery during mitral isthmus or CTI ablation, and (2) the remaining (*n* = 3, 12%) were generalized spasm. The latter represent the Prinzmetal’s type of spasm occurring after ablation anywhere within the LA. Three cases of proximity-related spasm occurred while intending to perform PFA of the left inferior PV, but fluoroscopy review revealed inadvertent anterior positioning of the pentaspline catheter in flower pose toward the mitral isthmus. Of the 25 cases of spasm, 2 (8%) culminated in ventricular fibrillation; both patients required resuscitation and nitroglycerin administration; however, both patients recovered.

Coronary spasm during AF ablation, although rare, has even been reported with RFA in proximity to the coronary arteries^31,32^. The increased depth of the electric field generated during PFA may increase this likelihood. Indeed, upon routine post-PFA coronary angiography, PFA at the CTI or mitral isthmus results in frequent subclinical coronary vasospasm^33,34^. And there have been case reports of patients manifesting clinical spasm^35,36^. Importantly, spasm is attenuated by pre-administration of intracoronary or intravenous nitroglycerin^33,34^. The possibility of ventricular fibrillation, although infrequent, suggests that nitroglycerin should be considered before PFA in proximity to coronary arteries.

Most intriguing are the cases of generalized spasm following PFA at locations remote from the coronary arteries. This appears to be a sympathetic/autonomic response to energy delivery, and is not unique to PFA. Indeed, in a meta-analysis of >22,000 Japanese patients, generalized coronary vasospasm during PVI occurred with both cryoablation (0.27%) and RFA (0.04%) at rates similar to that presently observed with PFA (0.017%; 3 of 17,640)^37^. Proceduralists must be aware of this rare complication as timely intervention is crucial in this circumstance.

An unexpected finding was hemolysis-related renal failure, which occurred in five patients (0.03%). Symptoms were reported either immediately post-procedure or by the next day, with rapid progression of oliguria or anuria, requiring dialysis for normalization in renal function. One or more factors additional to the higher number of PFA applications may have contributed, including dehydration, relative hypotension due to general anesthesia, contrast computed tomography on the day of procedure and some degree of pre-existing kidney disease. With thermal ablation, hemolysis-related renal failure is an extremely rare finding. In the surgical literature, an increased risk of acute kidney injury with concomitant surgical AF ablation has been reported, but the pathophysiology may not be the same^27^.

The mechanism of hemolysis is probably related to the electroporative effects on erythrocytes. Foundational experiments had demonstrated voltage-induced pore formation in human erythrocytes during therapeutic PFA applications^38^. Using clinical defibrillators, when a homogeneous electrical field (field strength of 1,700 V cm^−1^ as single or double monopolar or bipolar pulses) was applied to a human erythrocyte suspension, hemolysis occurred in a dose-dependent fashion^39^, and of course, the renal toxic effect of sudden high concentrations of globular hemeproteins is well appreciated (for example, myoglobinuria from rhabdomyolysis after traumatic crush injury to an extremity). In fact, there are probably more instances of under-recognized hemolysis not resulting in renal failure.

Given this putative mechanism, it is not surprising that all five patients received more complex lesion sets than simple PVI, with a mean of 143 PFA applications per patient; indeed two patients received 159 PFA applications. For context, in the PFA for persistent AF ablation (PersAFOne) trial, wherein patients underwent PVI, posterior wall isolation and CTI, an average of 46 PF lesions were applied per patient^19^. In a real-world retrospective analysis of 45 patients undergoing PVI, posterior wall ablation and mitral isthmus ablation, a total of 85 ± 23 PFA applications/patient were employed^40^. It seems likely that the risk of hemolysis is dose-dependent. Accordingly, it is prudent to moderate the number of PFA applications, and when a large number of PFA applications is necessary, one should consider applying simple mitigation strategies such as saline hydration.

The major complication rate was low at ~1% and primarily consisted of pericardial tamponade (0.36%), stroke (0.12%), and vascular complications (0.30%), with a procedural mortality rate of only 0.03%. Considering the novelty of the technology, diversity of operators, and the first utilization by most operators, these rates are consistent with an excellent safety profile. For comparison, in a large US registry of AF ablation between 2000 and 2010 including >90,000 patients, the rate of pericardial complications was 1.52%, stroke was 1.02% and mortality was 0.42% (refs. ^10,41^). Furthermore, all 24 sites who treated the initial 1,758 patients in the MANIFEST-PF safety study were also in MANIFEST-17K, and there was a striking learning curve observed with >50% reductions in arguably the two most important complications, namely pericardial tamponade (0.97% reduced to 0.43%) and stroke (0.39% reduced to 0.14%). Interestingly, these low complication rates were also observed in the remaining 82 expanded MANIFEST-17K sites, namely pericardial tamponade (0.30%) and stroke (0.11%); this is indicative that the learnings from the initial MANIFEST-PF experience (in other words, careful catheter manipulation with utilization of a J-tip guidewire and careful sheath management with diligent saline flushing) were successfully elaborated to the full community. This bodes well for future sites commencing utilization of PFA.

Finally, it is notable that, in a small subset of the MANIFEST-17K cohort who underwent routine post-procedural brain MRI, asymptomatic abnormalities were observed in only 9 of 96 patients (9.4%). Their clinical significance remains unclear, as asymptomatic MRI-detected brain lesions are commonly seen after conventional AF ablation and other interventional cardiac procedures and more recently with another PFA ablation catheter^42–44^. Indeed, this incidence compares favorably with the 26.1% rate of silent cerebral ischemic events observed during routine brain MRI in 321 patients undergoing RF or cryoballoon ablation in the prospective multicenter AXAFA-AFNET5 trial (anticoagulation using the direct factor Xa inhibitor apixaban during atrial fibrillation catheter ablation: comparison to vitamin K antagonist therapy)^45^

MANIFEST-17K is limited by being a retrospective observational study of center-level data without prospectively defined safety outcomes. However, most centers maintained a PFA registry from which the data were extracted, and the near-universal engagement of the centers for data acquisition (91.4% overall participation), the breadth of AEs reported and the sheer scale of PFA cases included (representing almost all post approval PFA cases for AF) extends credibility to the study. Second, it is possible that additional patients may have sustained subclinical events; examples include esophageal lesions that healed without symptomatology, asymptomatic PV stenosis, asymptomatic cerebral lesions, subclinical coronary spasm, hemolysis causing mild reversible renal dysfunction, and so on. Third, in the cases of suspected vasospasm, actual spasm was not always observed, probably because of both prompt nitroglycerin administration and the time delay to angiography; thus, clinical determination of spasm was based on a number of factors (for example, proximity of PFA location to a coronary artery, distribution of ST elevation, temporal response to nitroglycerin and so on). Fourth, while MANIFEST-17K included >400 operators at 106 sites, of which 38.7% were private practice and hybrid institutions, it is possible that this cohort of operators is enriched for greater expertize; accordingly, the favorable safety profile may not directly translate to all other centers. Finally, this study is focused on the pentaspline PFA catheter; because of potential variability between PFA technologies, the safety observed in this study should not be assumed for other PFA catheters.

In conclusion, this is the largest registry of the safety of the post-approval use of a PFA catheter for the treatment of AF. In a ‘real-world’ setting of an unselected AF patient population, PFA demonstrated a safety profile consistent with an important degree of preferentiality to myocardial tissue ablation, with no evidence of esophageal complications, and with a low rate of major complications. Hemolysis-related renal failure requiring hemodialysis did occur, albeit rarely. Finally, the low incidence of coronary arterial spasm belies its potentially serious implications and warrants further study and guidance.

## Methods

### Survey overview

The MANIFEST-17K study is a retrospective observational study of centers performing PFA after regulatory approval of the pentaspline PFA catheter (Farawave, Farapulse-Boston Scientific Inc). The data form was developed by two of the authors (E.E. and V.R.) with the goal of collecting comprehensive data on the methods and safety of the post-approval clinical use of PFA (Online Supplement, case report form pages 7–9). MANIFEST-17K was approved by the Homolka hospital ethical committee (6.4.2022/18). The requirement of informed consent was waived by the ethical committee given the restrospective nature of the study.

An invitation to participate in the MANIFEST-17K study was sent to all 116 centers performing clinical cases with this PFA catheter since commercialization. All centers who accepted the invitation were sent the comprehensive data form. Institution-level data were obtained on center characteristics, limited patient baseline characteristics, limited procedure parameters and all AEs. Additional root cause analysis data were obtained for specific AEs. Data were typically collected from each center’s institution-level ablation database when available. All data forms were provided with the condition of anonymity of the identity of the physicians and institutions. Of note, we excluded from this analysis the initial 1,758 patients treated in 2021 by the initial 24 centers (herein referred to as the ‘initial MANIFEST-PF sites’) and previously reported in the MANIFEST-PF survey (the ‘MANIFEST-PF cohort’). Thus, the patients included in this study include those patients treated after 2021 from the initial MANIFEST-PF sites plus all patients treated by newer sites not initially participating in MANIFEST-PF (the ‘expanded MANIFEST-17K sites’).

### The PFA procedure

The PFA system has been previously described; per manufacturer protocol, physicians were trained to employ a standard protocol^17–19^. Briefly, the 12F over-the-wire pentaspline PFA catheter (Farawave) is advanced through a 13F steerable sheath (Faradrive; Farapulse-Boston Scientific) into the LA. After positioning either a straight- or J-tip 0.035 guidewire into each target PV, the PFA catheter is positioned at the ostium of each PV and a total of eight PF lesions are applied per vein: four each in ‘basket’ and ‘flower’ configurations, with rotation between each pair of lesions. For posterior LA wall ablation, the catheter was placed into a flower configuration and positioned along the posterior LA to deliver overlapping sets of pulses at each location. The PF voltage amplitude could range between 1.8 and 2.0 kV, but 2.0 kV was typically employed. Unlike thermal ablation where one typically employs esophageal mitigation strategies (such as reduced ablation energy application along the posterior LA, esophageal temperature monitoring, esophageal cooling or mechanical esophageal deviation), no esophageal management strategy was employed during the PFA procedures.

### Study data specifics

The data form was composed of questions covering the following areas: geographic region, clinical site characteristics, baseline patient characteristics, procedural parameters and AEs (Online Supplement, case report form pages 7–9). Major complications were defined as death, stroke, esophageal fistula or dysmotility, PV stenosis, phrenic nerve injury (persistent), pericardial tamponade, vascular complications requiring intervention, coronary spasm, myocardial infarction, hemolysis with resultant renal failure requiring hospitalization/dialysis, and thrombosis. If a major AE was identified (specifically stroke and coronary spasm) a root cause analysis form or AE detail form, respectively, was sent to the clinical site. The root cause analysis collected information on the event details, the physician’s hypothesis as to the most likely etiology and recommendations to prevent future complications. Minor complications were defined as transient ischemic attack, phrenic nerve injury (transient), pericardial effusion (no intervention), vascular complications (no intervention), hemolysis not requiring hospitalization and others.

### Data analysis

The survey data form was considered complete if at least 80% of the questions were answered. In actuality, >95% of the forms were 100% completed. Importantly, the missing data were limited to the baseline patient demographics; there was no missingness in the reported safety outcomes.

Descriptive statistics were employed to analyze outcomes. Continuous variables were presented as means with minimum and maximum values provided. Continuous variables were compared using either the unpaired Student’s *t*-test (normal distribution) or Mann–Whitney *U* test (skewed distribution). Categorical variables were presented as counts or percentages and comparative analysis performed using the chi-squared or Fisher exact test. A two-tailed *P* value <0.05 was considered statistically significant. All analyses were performed using SPSS software (IBM Corp) version 29.

### Reporting summary

Further information on research design is available in the Nature Portfolio Reporting Summary linked to this article.

## Online content

Any methods, additional references, Nature Portfolio reporting summaries, source data, extended data, supplementary information, acknowledgements, peer review information; details of author contributions and competing interests; and statements of data and code availability are available at 10.1038/s41591-024-03114-3.

### Supplementary information

Supplementary Information Participating sites and MANIFEST-17K case report form.

Reporting Summary

## Figures and Tables

**Extended Data Table 1:**
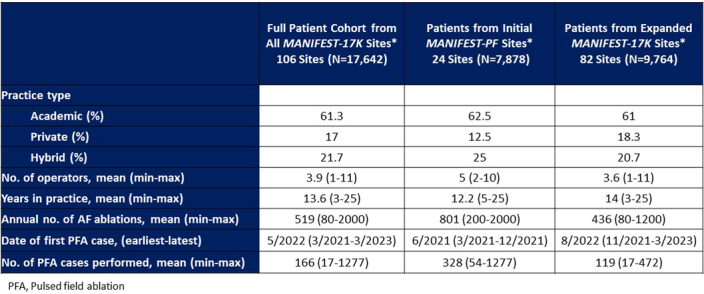
Clinical Site Characteristics

**Extended Data Table 2:**
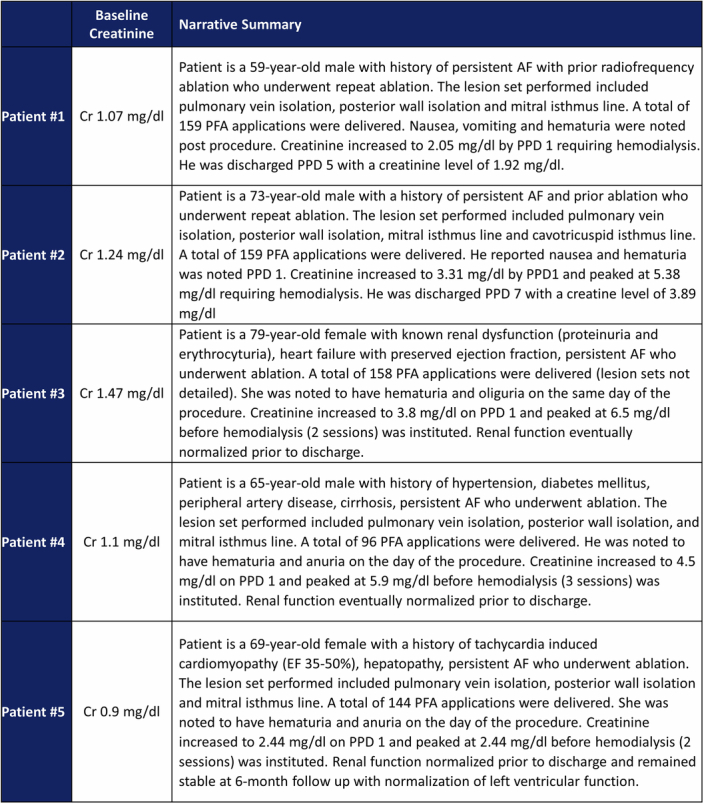
Hemolysis-Related Acute Renal Failure: Patient Details

**Extended Data Table 3:**
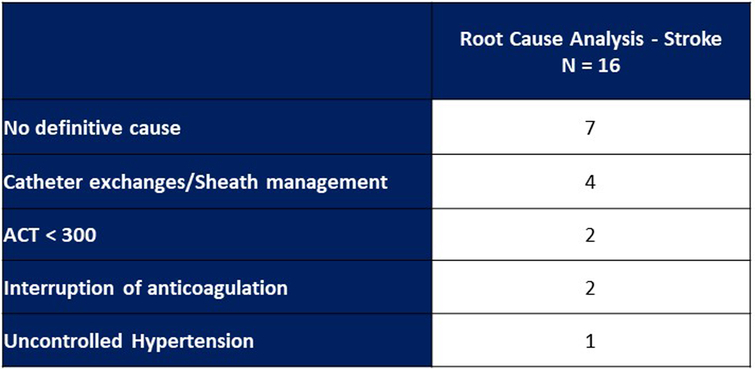
Root Cause Analysis of Stroke Events

**Extended Data Table 4:**
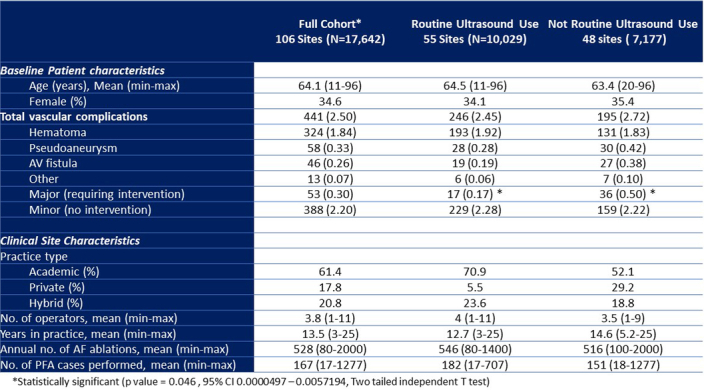
Vascular Complications as a Function of Routine Vascular Ultrasound Usage

**Extended Data Table 5:**
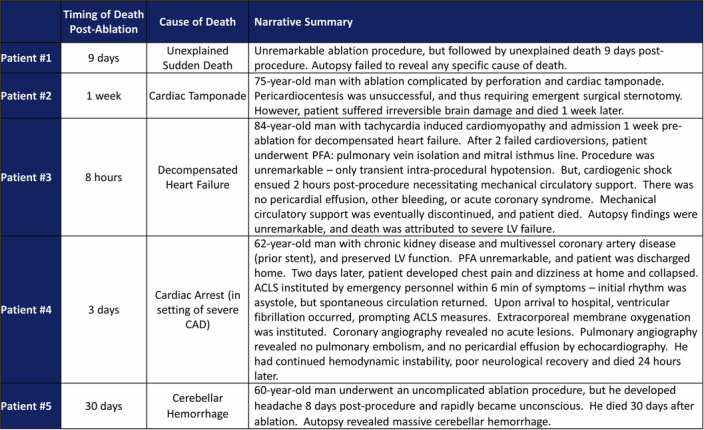
Details of Patient Deaths

**Extended Data Table 6:**
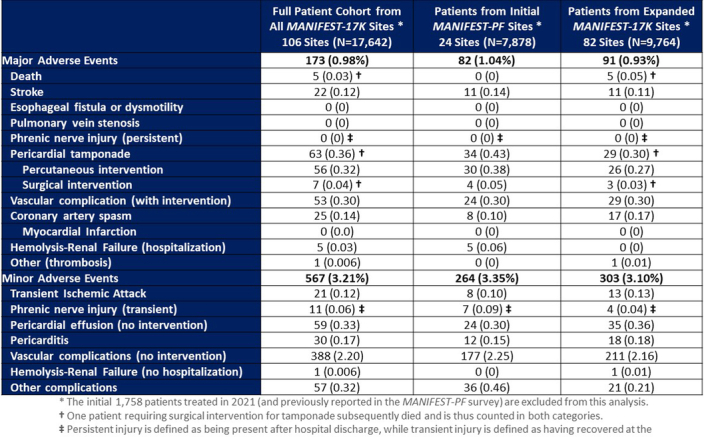
Complications in Both Cohorts – Initial *MANIFEST-PF* Sites *vs* Expanded *MANIFEST-17K* Sites

## Data Availability

Data can be made available upon reasonable request as part of a scientific collaboration with adherence to standards of good scientific practice. Restrictions may apply due to privacy reasons, scale of contributors and ongoing research projects. Requests should be sent to the corresponding author, and a period of 90 days should be expected for a response.
