# Supplementary material for: Safety of pulsed field ablation in more than 17,000 patients with atrial fibrillation in the MANIFEST-17K study
Source: Nat Med. 2024 Jul 8;30(7):2020–9. doi: 10.1038/s41591-024-03114-3 (PMC11271404; doi:10.1038/s41591-024-03114-3)
Supplement: Supplementary file 1 — Participating sites and MANIFEST-17K case report form. [file 41591_2024_3114_MOESM1_ESM.pdf]

nature medicine

Supplementary information

https://doi.org/10.1038/s41591-024-03114-3

Safety of pulsed field ablation in more than 17,000 patients with atrial fibrillation in the MANIFEST-17K study

In the format provided by the authors and unedited

Participating Centers

No.	Center, Location	Collaborators	No. of Patients
1	* Homolka Hospital, Prague, Czech Republic	Petr Neuzil, MD<br>Jan Petru, MD	1277
2	*Department of Cardiology, Inselspital, Bern University Hospital, University of Bern, Bern, Switzerland	Prof. Tobias Reichlin, MD<br>Thomas Küffer, MD	815
3	* IKEM, Prague, Czech Republic	Joseph Kautzner, MD<br>Petr Peichl, MD	707
4	* Catharina Ziekenhuis Eindhoven, The Netherlands	Pepijn van der Voort, MD<br>Lukas Dekker, MD	698
5	* IHU LIRYC, CHU Bordeaux, University of Bordeaux, France	Pierre Jais, MD	650
6	Heart Rhythm Management Centre, Postgraduate Program in Cardiac Electrophysiology and Pacing, Universitair Ziekenhuis Brussel, Vrije Universiteit Brussel, European Reference Networks Guard-Heart, Brussels, Belgium	Domenico G. Della Rocca, MD, PhD<br>Andrea Sarkozy, MD, PhD<br>Gian-Battista Chierchia, MD, PhD<br>Carlo de Asmundis, MD, PhD	472
7	Ceske Budejovice Hospital and Faculty of Health and Social Sciences, University of South Bohemia in Ceske Budejovice, Czech Republic	Alan Bulava, MD	460
8	*Universitair Medish Groningen, Netherlands	Yuri Blaauw, MD	457
9	University Hospital Olomouc, Czech Republic<br>Disclosures: none	Tomas Skala, MD, PhD<br>Ondrej Moravec, MD, PhD	441
10	*Neuron Medical, Brno, Czech Republic	Martin Fiala, MD, PhD<br>Moritoshi Funasako, MD	437
11	Az Sint Jan Hospital, Bruges, Belgium	Mattias Duytschaever, MD, PhD<br>Sebastien Knecht, MD, PhD<br>Rene Tavernier, MD<br>Benjamin De Becker, MD	435
12	1. Atrial Fibrillation Institute, Mater Private Hospital, Dublin Eccles street, Dublin 7, Ireland<br>2. Cardiovascular Research Institute, Royal College of Surgeons in Ireland	Prof. Gabor Szeplaki, MD, PhD<br>Gael Jauvert, MD	432
13	* Cardioangiologisches Centrum Bethanien, Frankfurt, Germany	Boris Schmidt, MD<br>Julian Chun, MD	403
14	Clermont Ferrand university hospital, 63000 Clermont-Ferrand, France	Grégoire Massoullie, MD<br>Romain Eschalier, MD, PhD	374

15	*Alfried Krupp Hospital<br>Dept. of Electrophysiologiy<br>Alfried-Krupp-Str. 21<br>45131 Essen<br>Germany<br><br>Witten/Herdecke University<br>Dept. of Medicine<br>Alfred-Herrhausen-Str. 50<br>58455 Witten<br>Germany	Kars Neven, MD, PhD<br>Anna Füting, MD	340
16	GHP Ambroise Paré Hartmann, neuilly sur Seine, France.	Olivier Thomas, MD<br>Alexandre ZHAO, MD	257
17	* Jessa Hospitals, Hasselt, Belgium	Johan Vijgen, MD<br>Pieter Koopman, MD	230
18	Sorbonne Université, APHP, Pitié-Salpêtrière Hospital, Institute of Cardiology, ICAN Institute for Cardiometabolism and Nutrition, Paris, France.	Estelle Gandjbakhch, MD<br>Mikael Laredo, MD	226
19	*Medical University of Graz, Austria	Daniel Scherr, MD<br>Martin Manninger, MD	220
20	*Copenhagen University Hospital Gentofte, Denmark	Arne Johannessen, MD, PhD<br>Jim Hansen, MD	218
21	Blackrock clinic, Dublin, Ireland	David Keane, MD<br>Daniel O'Hare, MD	214
22	*1- Heart Rhythm Management Department, Clinique Pasteur, Toulouse, France.<br>2- University of Brussels VUB, Jette Brussels, Belgium.	Serge Boveda, MD, PhD	210
23	*University Hospital Rangueil, Toulouse, France	Philippe Maury, MD<br>Anne Rollin, MD	205
24	1) Clinica Universidad de Navarra. University of Navarra. Pamplona, Spain<br><br>2) IdiSNA. Instituto de Investigación Sanitaria de Navarra. Pamplona, Spain	Ignacio García-Bolao , MD	200
25	*University Hospital Center Split, Split, Croatia	Ante Anic, MD<br>Zrinka Jurisic MD, PhD	194
26	privathospitalet mølholm a/s, Denmark	Peter Steen Hansen, MD	191
27	Clinique du Millenaire, Montpellier, France	Franck Raczka, MD	190
28	Centre Cardiologique du Nord, France	Antoine Lepillier, MD	190
29	GHICL Hôpital Saint Philibert, France	Yves Guyomar, MD	188
30	Liverpool Heart and Chest Hospital, Liverpool, UK	Dhiraj Gupta, MD	188

31	Enschede, Medisch Spectrum Twente, Netherlands	Jurren Van Opstal, MD	183
32	Cardiology Department, Grenoble Alpes University Hospital and University, 38043 Grenoble, France	Pascale Defaye,MD	180
33	University Hospital Basel, Switzerland	Christian Sticherling, MD	170
34	*Heart & Diabetes Center NRW, Ruhr-Univ Bochum, Germany	Philipp Sommer, MD<br>Thomas Fink, MD	169
35	Liberec, Czech Republic	Pavel Kucera, MD<br>Tomas Roubicek, MD, PhD<br>Jan Stros, MD	159
36	Polytechnic and University La Fe Hospital, Valencia, Spain.	Joaquin Osca, MD	157
37	Capio Arytmi center<br>Stockholm, Sweden.	Fariborz Tabrizi, MD, PhD.	153
38	pole sante republique elsan, France	Antoine Roux, MD	148
39	Uniklinikum RWTH Aachen, Department of Cardiology, Pauwelsstr. 30, 52074 Aachen, Germany	Michael Gramlich, MD	147
40	Ospedale Isola Tiberina Gemelli. Rome , Italy	Stefano Bianchi, MD	135
41	hospital de santa cruz, Portugal	Pedro Adragão, MD	134
42	Montevergine Clinic - Mercogliano (AV), Italy	Francesco Solimene, MD	130
43	Dept of Biomedical, Surgical and Dental Sciences, University of Milan, Milan, Italy	Claudio Tondo, MD	128
44	Biomedical Science and public Health Department UNIVPM Ancona Italy and Cardiology and Arrhythmology clinic Department Azienda Ospedaliera Universitaria Delle Marche Ancona, Italy	Antonio Dello Russo MD, PhD.	126
45	Medical University of Tuebingen, Germany	Jürgen Schreieck, MD	125
46	Städtisches Klinikum Karlsruhe, Germany	Armin Luik, MD	123
47	universitätsklinikum düsseldorf, Germany	Obaida Rana, MD	120
48	Clinic for Cardiology II - Electrophysiology, University of Münster, Germany	Gerrit Frommeyer, MD	120
49	*Rouen Hospital, Rouen, France	Frédéric Anselme, MD., PhD<br>Corentin Chaumont, MD	114
50	St.-Johannes-Hospital, Dortmund, Germany	Ingo Kreis, MD	110
51	Tel Aviv Sourasky Medical Center, Tel Aviv, Israel	Raphael Rosso, MD	110
52	*University Heart and Vascular Center, UKE-Hamburg, Germany	Andreas Metzner, MD	109
53	Semmelweis University, Cardiovascular Center, Budapest, Hungary	Laszlo Geller, MD	103
54	Hirslanden Klinik Beau-Site, Bern, Switzerland	Samuel H. Baldinger, MD	97

55	Hospital Clínico Universitario de Valencia, Spain	Angel Ferrero, MD	94
56	*Asklepios Hospital St.Georg, Hamburg, Germany	Stephan Willems, MD<br>Melanie Gunawerdene, MD	93
57	St. Vincenz-Hospital Paderborn, Germany	Andreas Goette, MD	93
58	Cardiology Dept. Royal Papworth Hospital, Cambridge, UK	Greg Mellor, MD<br>Claire Martin, MD	93
59	Universitätsklinikum Essen, Germany	Shibu Mathew, MD	90
60	National Institute of Cardiology, Warsaw, Poland	Lukasz Szumowski, MD, PhD	88
61	*Department of Rhythmology, University Heart Center Lübeck, University Hospital Schleswig-Holstein, Lübeck, Germany<br>German Center for Cardiovascular Research (DZHK), Partner Site Lübeck, Germany	Roland Tilz, MD<br>Bettina Kirstein, MD	86
62	Maria Cecilia Hospital, Cotignola, Italy	Saverio Iacopino, MD	86
63	Rigshospitalet, University Hospital of Copenhagen, Denmark	Peter Karl Jacobsen, MD<br>Niels Sandgaard, MD	80
64	Henry Dunant Hospital, Athens, Greece	Andrikopoulos George MD, PhD, FESC,	80
65	Fnkv, Czech Republic	Pavel Osmancik, MD, PhD	79
66	Praxisklinik Herz und Gefäße, Dresden, Germany	Stefan Spitzer, MD	79
67	Nhssc Royal Bournemouth Hospital, UK	Richard Balasubramaniam, MD	76
68	Deutsches Herzzentrum der Charité, Campus Virchow-Klinikum, Germany	Abdul Shokor Parwani, MD	74
69	*Heart Center Bad Neustadt, Germany	Thomas Deneke, MD<br>Karin Nentwich, MD	71
70	Department of Cardiology, Medical University of Lublin, Poland	Andrzej Glowniak, MD, PhD<br>Aleksander Konopka, MD<br>Adam Tarkowski, MD<br>Katarzyna Wojewoda, MD	71
71	Department of Cardiology, San Bortolo Hospital, Vicenza, Italy	Antonio Rossillo, MD	70
72	Ordensklinikum Linz Elisabethinen, Linz, Austria	Helmut Pürerfellner, MD	65
73	Hannover Heart Rhythm Center, Department of Cardiology and Angiology, Hannover Medical School, Hannover, Germany	David Duncker, MD	65
74	Klinikum Ingolstadt, Germany	Peter Reil, MD<br>Vjosa Thaci-Kadriu, MD	64
75	*Universitätsklinikum Freiburg, Germany	Thomas Arentz, MD<br>Heiko Lehrmann, MD	63

76	*Universitätsklinikum Köln AöR, Germany<br>Department for Electrophysiology, Heart Center University Cologne, Cologne, Germany.	Daniel Steven<br>Arian Sultan, MD	58
77	Arrhytmia Service, Cardiology Department, University Hospital Marqués De Valdecilla, Santander, Spain	Juan José Olalla, MD, PhD<br>Felipe Rodriguez-Entem, MD, PhD<br>Víctor Exposito, MD,PhD	58
78	OLVG, Amsterdam, The Netherlands	Jonas S.S.G. de Jong, MD, PhD	56
79	*University Duisburg-Essen, Duisburg, Germany<br>University Hospital Frankfurt, Goethe University Frankfurt, Germany	Reza Wakili, MD<br>Jan Bohnen, MD	54
80	L'Hôpital Privé du Confluent, Nantes, France	Selim ABBEY, MD	50
81	Christliches Klinikum Unna, Unna, Germany	Gottschling Timo MD<br>Georg Nölker, MD	50
82	Hospital Quironsalud, Zaragoza, Spain.	Antonio Asso, MD	50
83	Royal Brompton and Harefield Hospitals, Guy's and St Thomas' NHS Foundation Trust. King's College and Imperial College, UK.	Tom Wong, MD	50
84	Chru Tours Hôpital TROUSSEAU, France.	Bertrand Pierre, MD	48
85	Vivantes Klinikum Am Urban, Berlin, Germany	Niels Christian Ewertsen, MD	47
86	Georg-August-Universität Göttingen, Germany	Leonard Bergau, MD<br>Nibras Soubh	47
87	Arrhythmia Unit, Cardiology Department, University Hospital Ramón y Cajal, Madrid, Spain	Cristina Lozano-Granero, MD PhD<br>Javier Moreno Planas, MD	47
88	Ziekenhuis Oost Limburg, Genk, Belgium	Maximo Rivero, MD, PhD	46
89	kantonsspital baden ag, Switzerland	Alexander Breitenstein, MD	46
90	Tampere Heart Hospital, Tampere University Hospital, Finland	Jaako Inkovaara, MD	44
91	Hopital De La Croix Rousse Nord, France	Samir Fareh, MD<br>Paul Charles, MD<br>Mathieu Montoy, MD	43
92	Centre Hospitalier Princesse Grace, Monaco	Decebal Gabriel Latcu, MD	41
93	1) Department of Cardiology, Cardiovascular Research Institute Maastricht (CARIM), Maastricht University Medical Centre, Maastricht, The Netherlands.<br>2) Faculty of Health and Medical Sciences, Department of Biomedical Sciences, University of Copenhagen, Copenhagen, Denmark.	Dominik Linz, MD, PhD	38
94	Knappschafts-Kh Recklinghausen, Germany	Patrick Müller, MD	37

95	Arrhythmias Unit. Cardiology Department, Lozano Blesa Clinical University Hospital, Zaragoza, Spain. Aragón Health Research Institute, Zaragoza, Spain.	Javier Ramos-Maqueda, MD	33
96	Heart Center Bonn, Department of Internal Medicine II, University Hospital Bonn, Bonn, Germany	Thomas Beiert, MD<br>Jan Schrickel, MD	30
97	*Ospedale dell'Angelo*, Mestre-Venezia, Italy	Sakis Themistoclakis, MD	30
98	Carl-Thiem-Klinikum Cottbus, Germany	Dirk Grosse Meininghaus, MD	29
99	Allgemeines Krankenhaus Universitätsklinik Wien, Austria	Günter Stix, MD<br>Robert Schonbauer, MD	28
100	Mitera Hospital, Athens Greece	Stylianos Tzeis, MD	26
101	Department of Internal Medicine and Cardiology University Clinical Center, Medical University of Warsaw, Warsaw, Poland.	Jakub Baran, MD, PhD.	26
102	Linköpings University Hospital, Sweden	Henrik Almroth, MD	23
103	Hospital Doce De Octubre, Spain	Daniel Rodriguez Munoz, MD, PhD	22
104	Arrhythmia Unit. Cardiology Department. Santa Maria University Hospital. Lisbon Academic Medical Center, Portugal	João de Sousa, MD	21
105	Onassis Cardiac Surgery Center Department of Cardiac Electrophysiology and Pacing, Athens, Greece	Michalis Efremidis, MD	18
106	Uniwersyteckie Centrum Kliniczne, Warsaw, Poland	Pawel Balsam, MD, PhD	17

***** Sites which also participated in the initial *MANIFEST-PF* study.

MANIFEST-17K CRFs

MANIFEST-17K: Multi-national Survey on Methods, and Safety on the Post-approval Clinical use of Pulsed Field Ablation

[*Case Report Form; Note: all below questions refer to the Farapulse Farawave Catheter*.]

List Center: ____

1. Region: Which country is your center from? __________________________

2. Demographics/Practice:
   a. How many different operators at your center perform pulsed field ablation?

      ____

   b. Years in practice of the electrophysiologist(s)

      ____

   c. Approximate annual number of AF ablations performed at your center

      ____

   d. Practice "type": Academic, Private, Hybrid

      ____

3. When was commercial pulsed field ablation introduced at your center (month/year)?

   ____

4. For data provided below, what are the dates of the first and last procedures performed (month/year)?

   First: __________________________ Last: __________________________

5. How many pulsed field ablation procedures have been performed at your center for atrial fibrillation/flutter/tachycardia (for *MANIFEST-PF* Consortium sites, please do not include any patients reported in the initial *MANIFEST-PF* Safety Evaluation)?
   (please complete in table below)

Total number of patients undergoing PFA for AF/AFL/AT	
How many patients with Paroxysmal AF?	
How many patients with persistent AF?	

How many patients with long standing persistent AF?	
How many patients with atrial flutter/tachycardia ***only***?	

6. What are the baseline characteristics of the patients who have undergone pulsed field ablation (for AF only) at your center? (please complete table below)

Age (average, minimum, maximum)	AVG:_______ MIN: ______ MAX:_______
Sex (male=n, female=n)	MALE:_________ FEMALE:_________

7. Typical anesthesia strategy:

a. Fraction of procedures using GA (with endotracheal intubation): ______%

b. Fraction of procedures using deep sedation (no intubation): ______%

8. Complications after pulsed field ablation: please complete table below.

**Complications**	n
Peri-procedural Death (within 30 days)	
Myocardial Infarction (within 30 days)	
Phrenic nerve injury<br>---Total number of events<br>---Number recovered be next day<br>---Number recovered by 1 month<br>---Not yet recovered (list longest f/u)	 ______________________________<br>______________________________<br>______________________________<br>______________________________
Stroke (within 7 days)	
TIA (within 7 days)	
Silent cerebral events (if screening MRIs done)<br>---Number of pts with MRI<br>---Number with positive MRIs	 ______________________________<br>______________________________
Other thromboembolism	
Pericardial effusion<br>---Total number of events<br>---No intervention<br>---Requiring ***percutaneous*** intervention<br>---Requiring ***surgical*** intervention	 ______________________________<br>______________________________<br>______________________________<br>______________________________

Pericarditis requiring intervention (eg, steroids, colchicine-not prophylactic)	
Vascular complications<br>---Total number of events<br>---hematoma<br>---pseudoaneurysm<br>---A-V fistula<br>---Other (please specify)<br>---How many required intervention?<br><br>---Is it routine practice to use vascular ultrasound to guide femoral puncture??	 ______________________________<br>______________________________<br>______________________________<br>______________________________<br>______________________________<br>______________________________<br><br><br>Yes / No /Other _______________
Pulmonary Vein stenosis	
Atrio-esophageal fistula<br>---requiring surgery<br>---causing death	______________________________<br>______________________________<br>______________________________
Esophageal/Gastric Dysmotility	
Coronary artery injury/spasm	
Other complications (please specify)	

Please provide any details for any of the above complications (especially for any esophageal complications, PV stenosis, phrenic nerve paralysis, stroke, coronary spasm, death, unusual events):

____
____
____
____
____
____
____
____
____
____
____
____
____
____
____
____
____
____
